# Critical Considerations in the Interpretation of Bone Turnover Marker Data in Hormonal Contraceptive Users. Comment on Tassi et al. Hormonal Contraception and Bone Metabolism: Emerging Evidence from a Systematic Review and Meta-Analysis of Studies on Post-Pubertal and Reproductive-Age Women. *Pharmaceuticals* 2025, *18*, 61

**DOI:** 10.3390/ph18091401

**Published:** 2025-09-18

**Authors:** Jonathan Douxfils, Jean-Michel Foidart

**Affiliations:** 1 Clinical Pharmacology and Toxicology Research Unit, Namur Research Institute for Life Sciences (NARILIS), University of Namur, 5000 Namur, Belgium; 2 Qualiblood s.a, QUALIresearch, 4000 Liège, Belgium; 3 Department of Biological Hematology, Centre Hospitalier Universitaire Clermont-Ferrand, Hôpital Estaing, 63100 Clermont-Ferrand, France; 4 Department of Obstetrics and Gynecology, University of Liège, 4000 Liège, Belgium

**Keywords:** bone turnover markers, hormonal contraceptives, bone mineral density (BMD), estrogen and bone health, adolescent skeletal development, combined hormonal contraceptives (CHCs)

## Abstract

In response to the recent meta-analysis by Tassi et al. on hormonal contraception and bone metabolism, we raise critical concerns regarding the interpretation of bone turnover markers as surrogates for bone mineral density (BMD). While bone turnover markers can offer early insights into bone remodeling, they do not directly predict long-term BMD changes, which require 12–24 months to detect. The assumption that equivalent percentage changes in bone formation and resorption markers reflect stable BMD is not supported by current evidence. Bone metabolism varies significantly across life stages, particularly during adolescence and early adulthood, when peak bone mass is still accruing—underscoring the need for age-specific analyses. Additionally, biomarker interpretation is limited by assay variability, inconsistent sampling protocols, and uncertain clinical implications, especially for formation markers. Mechanistically, estrogen supports bone integrity by inhibiting resorption and promoting formation; thus, combined hormonal contraceptives (CHCs) containing estrogen may help preserve bone health. In contrast, progestin-only methods can suppress endogenous estrogen production, potentially compromising skeletal development. We advocate for longitudinal studies incorporating both BMD and turnover markers, stratified by age and contraceptive formulation, to guide safer and more informed contraceptive choices that protect long-term bone health.

We read, with interest, the article titled “Hormonal Contraception and Bone Metabolism: Emerging Evidence from a Systematic Review and Meta-Analysis of Studies on Post-Pubertal and Reproductive-Age Women” [1]. In this study, the authors focused on bone marker changes rather than on bone mineral density (BMD). However, we have significant concerns regarding both the methodology and the conclusions drawn in the paper.

While there are several reports recommending bone turnover markers, especially C-terminal telopeptide (CTX) and N-terminal propeptide of procollagen type I (PINP), as appropriate endpoints in evaluating bone turnover, often used complementary to BMD measurement after drug intervention [2,3], the evaluation and comparison of bone-turnover markers results should be done carefully and with limitations in mind.

The authors conclude that the effect of combined hormonal contraceptives (CHCs) on bone health is beneficial when the percentage decrease in markers of bone resorption is greater than the decrease in bone formation markers. This assumption, however, relies on the premise that identical percentage changes in markers of bone formation and resorption would result in no change in BMD. As we discuss below, this assumption is flawed, and we highlight critical concerns regarding the interpretation of bone turnover marker changes in relation to BMD.

The concentration of bone turnover markers in plasma/serum or in urine reflects bone remodeling activity and can potentially serve as surrogate markers of the rate of bone formation or resorption [4]. While changes in these markers accurately reflect fluctuations in bone metabolism, the simple addition of bone resorption and bone formation markers do not provide a direct measure of BMD changes [3,5].

The authors assume that a 20% decrease in resorption markers (CTX1) and a 20% increase in bone formation markers (P1NP or osteocalcin) would result in a stable BMD. This assumption is in fact incorrect. They claim that CHCs with a higher impact on one marker of bone formation and a lower impact on one marker of bone degradation is preferable. To reach that conclusion, there should be an equal linear correlation between BMD changes and changes in those markers. This is particularly important considering that CHCs are widely used by young women, a population in which long-term bone health is critical [6]. Misinterpretation of these findings could influence prescribing practices, potentially leading to unintended consequences on peak bone mass accumulation.

Biochemical markers of bone turnover complement BMD measurements, but do not replace them. While bone turnover markers show early responses to treatment, their high within-person variability limits their predictive value for long-term BMD changes [5]. BMD changes following treatment require 12–24 months for reliable detection, making short-term marker fluctuations an unreliable proxy for long-term bone health [4]. However, given the dynamic nature of bone metabolism across different life stages [7], there is a critical need for age-specific studies to better define normative bone turnover patterns and treatment responses. This is particularly relevant for understanding the impact of CHCs on peak bone mass in adolescents and young adults, as hormonal modulation during this crucial period may have lasting effects on long-term skeletal health. Importantly, as correctly noted in the meta-analysis of Tassi et al., age-stratified results revealed notable differences in bone turnover marker response. For example, EE-containing COCs showed a larger decrease in osteocalcin in adolescents (SMC −0.63) than in women over 21 (SMC −0.42). Similar patterns were observed for other markers such as CTX1. These findings underscore that pooling adolescent and adult data without age stratification may introduce bias, particularly when studying interventions affecting peak bone mass accrual.

Additionally, while elevated bone resorption markers have been associated with increased fracture risk in elderly women [8], the relationship between bone formation markers and fracture risk or peak bone mass remains unclear. The clinical use of bone turnover markers for fracture prediction requires additional standardization concerning the time of collection of biological samples, choice of markers, expression of urinary markers, and definition of the clinically valid thresholds, as well as the choice of type of fracture and the duration of the follow-up for which bone turnover markers may be valid. Importantly, bone turnover marker changes do not reliably predict BMD responses to bisphosphonates, menopausal replacement therapy, or CHCs [9,10]. Single marker measurements fail to predict BMD cross-sectionally or longitudinally in both treated and untreated patients. The bone remodeling process is governed by a dynamic coupling between osteoclast-mediated resorption and osteoblast-mediated formation.

Several studies have demonstrated that this physiological coupling can be disrupted by hormonal influences, particularly in states of estrogen deficiency or suppression. Consequently, the assumption that symmetrical changes in bone turnover markers (BTMs) equate to stable BMD oversimplifies this complex physiology and is not supported by longitudinal studies investigating BTM–BMD relationships [2,4,5].

Estrogens play a pivotal role in bone homeostasis by inhibiting osteoclast-mediated resorption and promoting osteoblast survival, largely through modulation of the RANK/RANKL/OPG signaling pathway and suppression of pro-resorptive cytokines such as IL-1 and TNF-α [11]. Progestin-only contraceptives can reduce endogenous estrogen production [12,13], and evidence on their impact on BMD and fracture risk, particularly in adolescents and young women who have not yet reached peak bone mass, remains limited. Reflecting this uncertainty, product labeling for several progestin-only contraceptives includes warnings about potential negative effects on bone health, linked to decreased endogenous estrogen levels and unknown long-term consequences for BMD and fracture risk [14,15]. In contrast, CHCs supply exogenous estrogen, helping maintain more physiological estrogen concentrations and thereby supporting bone integrity.

Crucially, the dose and timing of estrogen exposure must be considered alongside the developmental stage at initiation. Hadji et al. [16] have argued that 4 mg drospirenone (DRSP) preserves bone protection by sustaining endogenous estradiol concentrations of 35–50 pg/mL, a range adequate for bone maintenance in postmenopausal women. However, this cannot be extrapolated to adolescents, where the objective is not merely to maintain but to build peak bone mass. During puberty, physiological estradiol rises progressively from mid-pubertal levels (~40–50 pg/mL) to adult values (~100 pg/mL), driving maximal bone mineral accrual. The Endocrine Society’s recommendations for pubertal induction in hypogonadal adolescents advocate gradual dose escalation over 2–3 years to mimic this natural progression, optimize bone accrual, and avoid premature epiphyseal closure [17]. A fixed, low endogenous estradiol level induced by progestin-only contraceptives may therefore be inadequate for optimal skeletal development in this age group.

Beyond circulating hormone levels, the type of estrogen in a contraceptive formulation influences bone and systemic effects through differences in molecular structure, estrogen receptor binding, and tissue selectivity. Estradiol (E2), the endogenous ligand, binds both ERα and ERβ with high affinity, activating genomic and non-genomic pathways essential for bone metabolism [18]. Ethinylestradiol (EE), a synthetic analogue with an ethinyl group at C17α, has high oral potency and strong ERα-mediated hepatic stimulation, resulting in pronounced effects on liver protein synthesis [19]. Estetrol (E4), a native fetal estrogen, binds both ERα and ERβ but behaves as a natural estrogen with selective tissue activity (NEST), showing full agonism in bone and endometrium while exerting weaker or antagonistic effects in tissues such as the breast [20]. Preclinical evidence indicates that E4 retains antiresorptive bone effects while producing less hepatic stimulation of coagulation factors, potentially offering a favorable balance between skeletal benefit and thrombotic safety [21,22]. Accordingly, interpretation of BTM or BMD data should integrate not only estrogen dose and timing but also receptor selectivity and tissue-specific pharmacodynamics, which can differ substantially among EE, E2, and E4.

Moreover, individual variability in bone turnover marker responses to CHCs must be considered, as genetic, lifestyle, and dietary factors may influence outcomes [2]. Generalizing conclusions from pooled data without accounting for these factors could lead to misrepresentation of the true effects of CHCs on bone health.

Additional points that should be considered when presenting bone marker data from different studies are (1) the fact that the sample collection and assays used in the different studies are not standardized (even for the same biomarkers), which could have a significant impact on the final results; (2) not all biomarkers were measured in all combinations presented, and therefore the pooling may be influenced by the higher impact of one marker versus another; and (3) as previously stated, effects on bone health and BMD cannot be reduced to a simple addition of bone formation and resorption markers, and presenting them as such is misleading and scientifically incorrect.

While bone turnover markers remain valuable for research applications in bone disease pathogenesis and treatment evaluation, their role in predicting BMD changes and fracture risk in young populations requires further validation. Importantly, the interpretation of bone turnover marker results on their own should be done with all the limitations in mind and with critical consideration of the limitations of inter-dataset comparison.

Given these uncertainties, future research should focus on longitudinal studies incorporating both BMD and bone turnover markers to assess the true impact of CHCs on bone health. Such studies should also consider variations between different estrogen-progestin combinations to provide more accurate recommendations for clinical practice.
